# Supplementary material for: Modular Micro Raman Reader Instrument for Fast SERS-Based Detection of Biomarkers
Source: Micromachines (Basel). 2022 Sep 22;13(10):1570. doi: 10.3390/mi13101570 (PMC9610109; doi:10.3390/mi13101570)
Supplement: Supplementary file 1 [file micromachines-13-01570-s001.zip › micromachines-1920863-supplementary.pdf]

SUPPORTING INFORMATION

Modular Micro Raman Reader instrument for fast SERS-based detection of biomarkers.

Jamison Duckworth [1] and Alexey V. Krasnoslobodtsev [1,2,*]

1 Department of Physics, University of Nebraska Omaha, Omaha, NE 68182, USA

2 nDETKT, LLC.

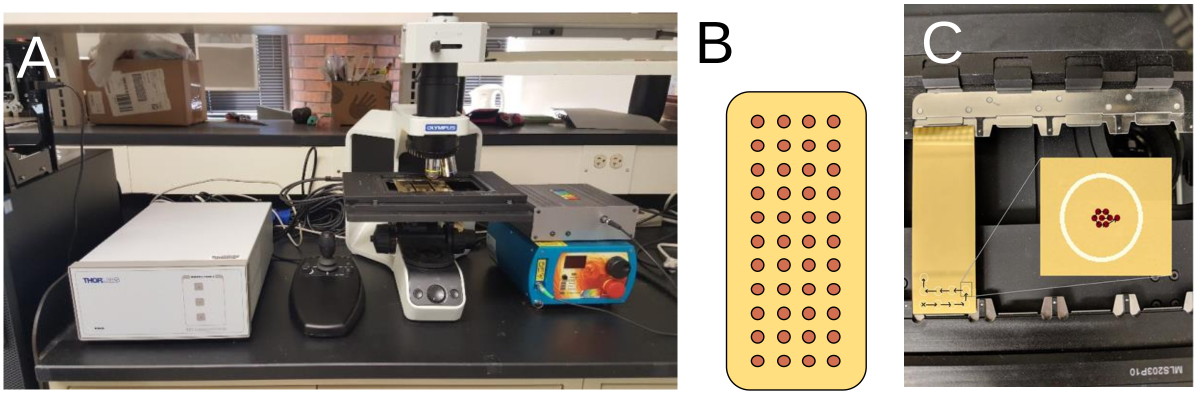

Figure S1. Constructed micro Raman Reader instrument. **(A)** improved for automated measurements: motorized x-y stage with 4 slide capability (concurrently). Components of the system: computer, stage motion controller, joystick, microscope frame, x-y motorized piezo stage, optical path: objective (long working distance), coupling, fiber optic cable, Raman spectrometer, laser (647 nm excitation). **(B)** Detection chip layout with 48 addresses of individual samples. **(C)** scanning pattern on the wafer (left – raster scan) and on an address (right – vector scan) – see section 1 below for details.

1. Movement Method

The algorithm for stage translation, which is the primary method for data acquisition, has three basic parts: wafer movement, address movement, and inner address movement. The program is designed for a maximum of 4 gold slides (or wafers) with similarly deposited samples for analysis. The design was tailored for the MLS203 x-y motorized piezo stage from Thorlabs, Inc. equipped with MLS203P10 Multi Slide Holder.

Wafer movement is the simplest one out of all in the code. Here a *for* loop with a maximum value of 4 carries out the movement across the wafers due to the maximum number of slides used. A button selection on the Graphical User Interface (GUI) of the LabView interface sets the number value to *true* if selected. This is indicated by a green light on the button below the selected wafer. If a slide is not selected it will remain *false* and will skip over that wafer during the wafer movements iteration of that slide. This part of the GUI is shown at the bottom left side of Figure S2.

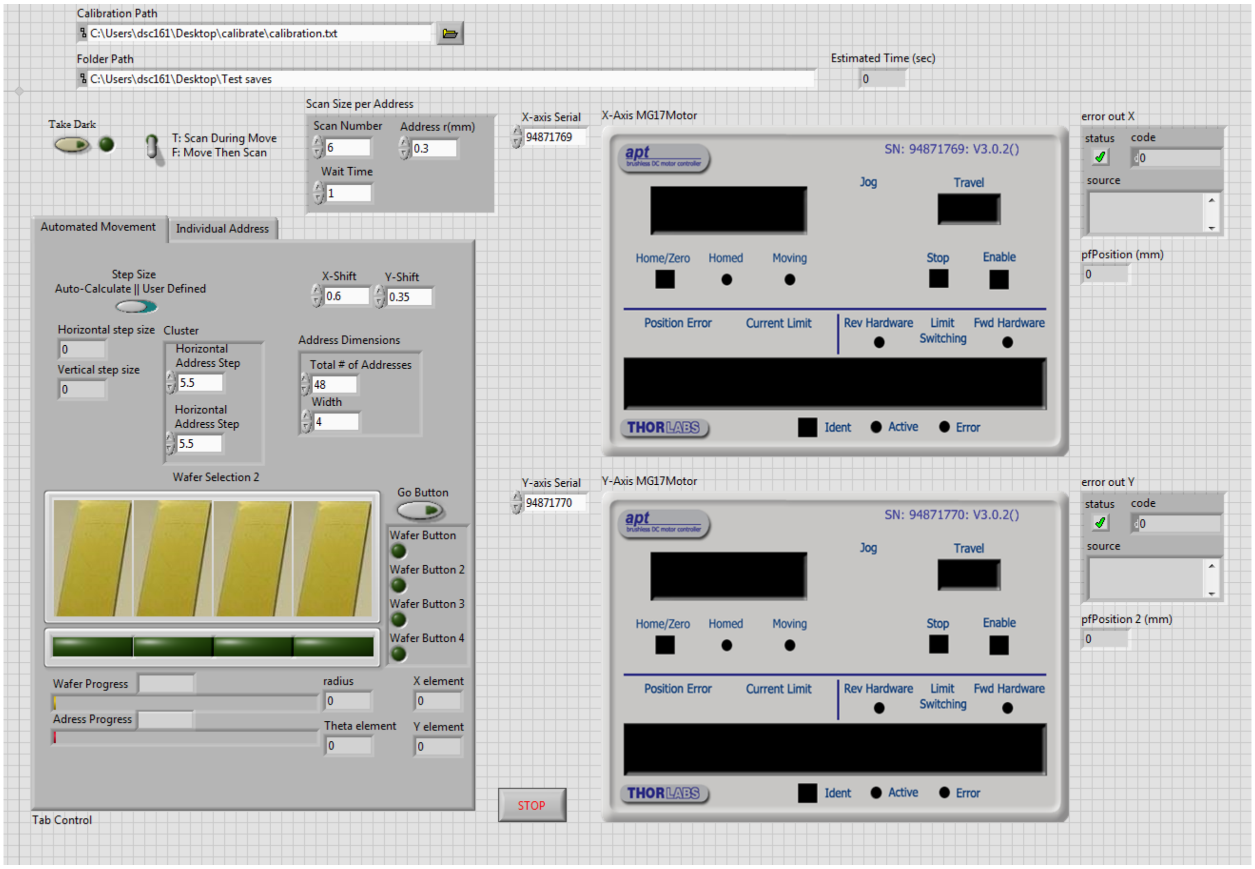

Figure S2: GUI control for stage translation. Gold wafers are buttons, and the green lights will light up when the buttons are toggled on.

After the movement to the selected wafer, the code then moves across each element of the address. It is assumed that the wafers used are 25 x 75 x 1mm slides and that the addresses are evenly distributed (in x and in y). This assumption allows for the automation of address scanning. Referring to Figure S2, just above the wafer buttons is a recessed "Address Dimensions" panel. Here, the user can choose the total number of addresses on the wafer and how many span the width, labeled "Total \# of Addresses" and "Width" respectively. The width is defined as the number of addresses across the shortest distance of the wafer.

Once entered, the code will send the values to a sub-routine for the inner wafer movement. Here a *for* loop is used to make an array of values for the 2-dimensional stage to use. This is depicted in Figure S3 with the number of addresses as the end value for the *for* loop and the width working as a step counter (0's and 1's). A flip is needed to move back and forth for every other row, which is located under the stepping method, and a horizontal shift is also needed to move to the next row, which is under the flipping method. This resulting pattern is that of a raster scan method. The array then gets passed to the stage along with a dwell time (in Figure S2, labeled as "Wait Time").

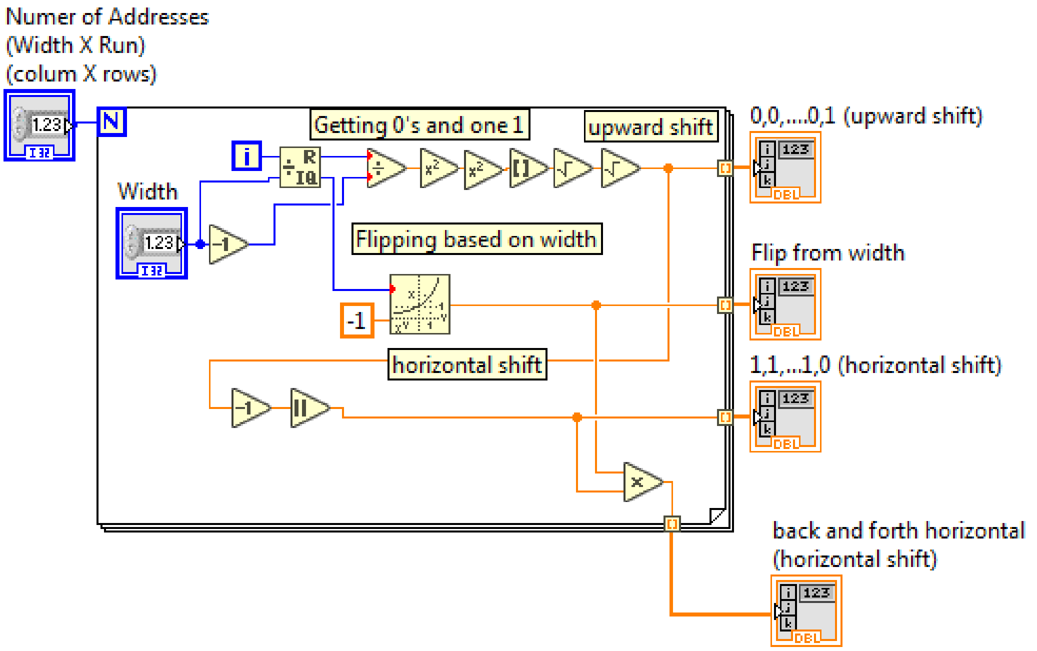

Figure S3: Address movement module. The code sends array to the x-y stage based on the address's width and maximum number.

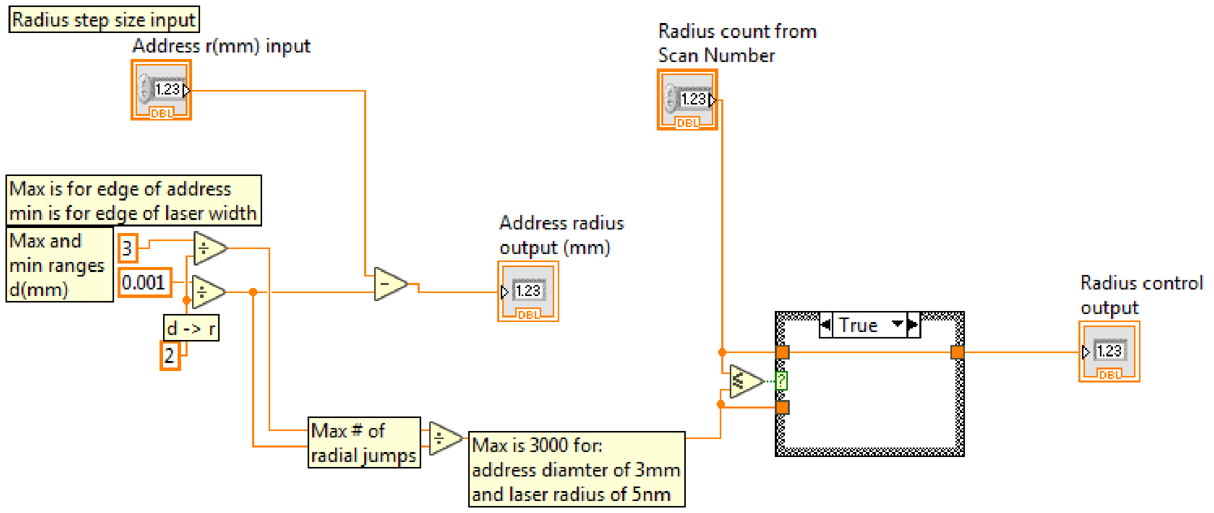

Figure S4: Radius control module.

Next, the inner address method is defined by mapping out concentric rings and relaying them to the stage translation. This is done in two parts. First is the radius control, shown in Figure S4, that calculates the address radius in millimeters and feeds it to the radius counter for pattern manipulation (Figure S5).

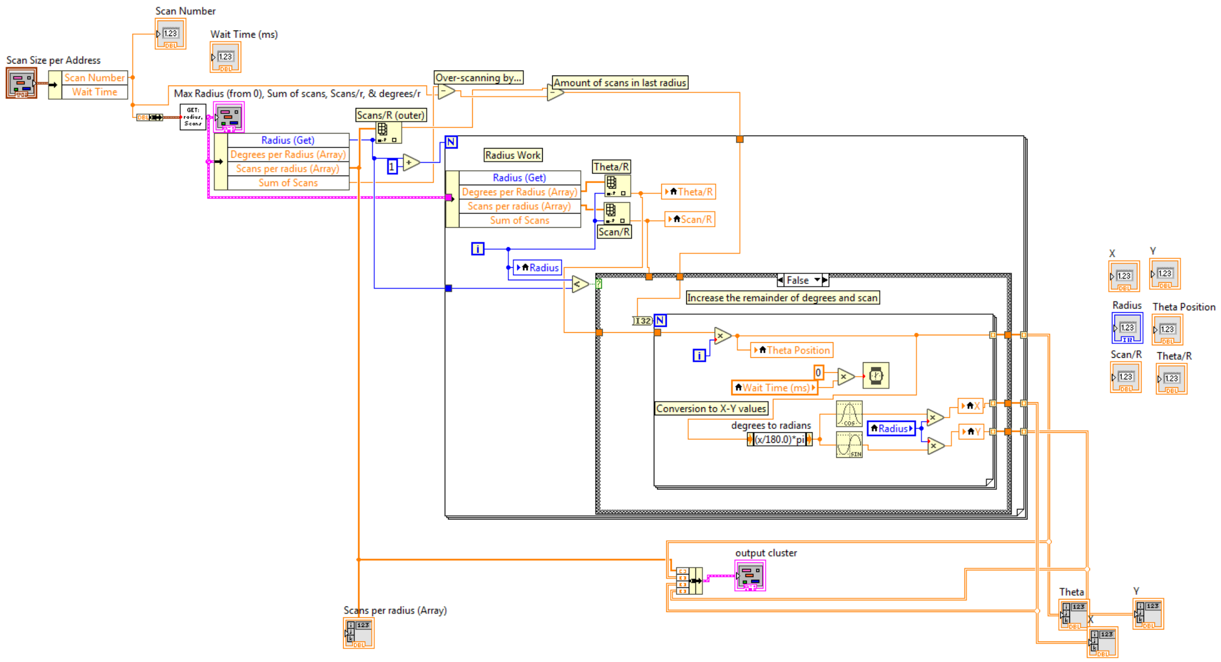

Figure S5: Radius counter module. Inputs enter at top of page and passable outputs are at bottom of page. The for loop is to keep the array order and the true/false conditional loop (inside the for loop) controls whether there is circular movement or not. If the conditional loop is true, it is the final loop.

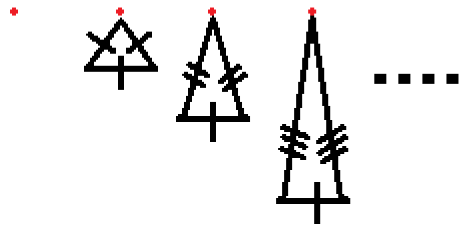

Figure S6: Triangle pattern for the concentric ring calculation. Red dot is the first scan. Cross lines represent number of diameters out.

In Figure S5 is a hard-coded pattern for inner address pattern. That is to say that the concentric ring calculation was done on paper and then made into LabView code. The method here is to scan in laser diameter-unit increments for the radius for pattern progression. For the first area there will be 0 diameter and only one scan. For the second area there are 6 places to scan that correspond to six vertices on a regular hexagon. This is due to going a diameter away from the first scan and repeating at a diameter-regular intervals around the first scan. The third area goes out two diameter lengths from the center scan and will do this until it matches or breaks 360 degrees. Once it matches or breaks that it will move onto the next concentric circle, 3 diameters out, and continue this pattern until either the desired number of scans are met or the maximum address radius is equal to or less than the diameter away from the center scan. This pattern results in a vector-scan method for concentric circles that are on the vertices of touching isosceles triangles. For visualization see Figure S6.

After the code has moved the THORLABS stage to the appropriate wafer, then moved to the appropriate address (Figure S3), and after the concentric spots have been imaged (Figure S4 and S5) the code returns to its value at the beginning of the inner address movement in order to move to the next address. This is to prevent drifting. Then, after all the addresses have been imaged with the inner address specifications, the code redirects the value for the next wafer and repeats for the number of wafers selected. Lastly, once the pattern has been completed the program will stop and return to home.

Thus, through a 3-step program this code acquires translation of a stage for data acquisition. The first step being a simple shift for what wafer is desired, then into a raster-scan for moving between addresses and ends in a vector-scan around a concentric circles that are outlined by increasing isosceles triangles.

2. Control Method

The over-arching control for the code starts as a simple stage control. First, it is necessary to get the stepper motor addresses and wire them so that LabView can pass data back and forth. After this has been done the code continues into the main while loop that centers the stage,

passes data back and forth to the SpectraWiz software for Raman Spectroscopy analysis, and execution of the above algorithm. Full while loop is depicted in Figure S7 (spans 3 pages).

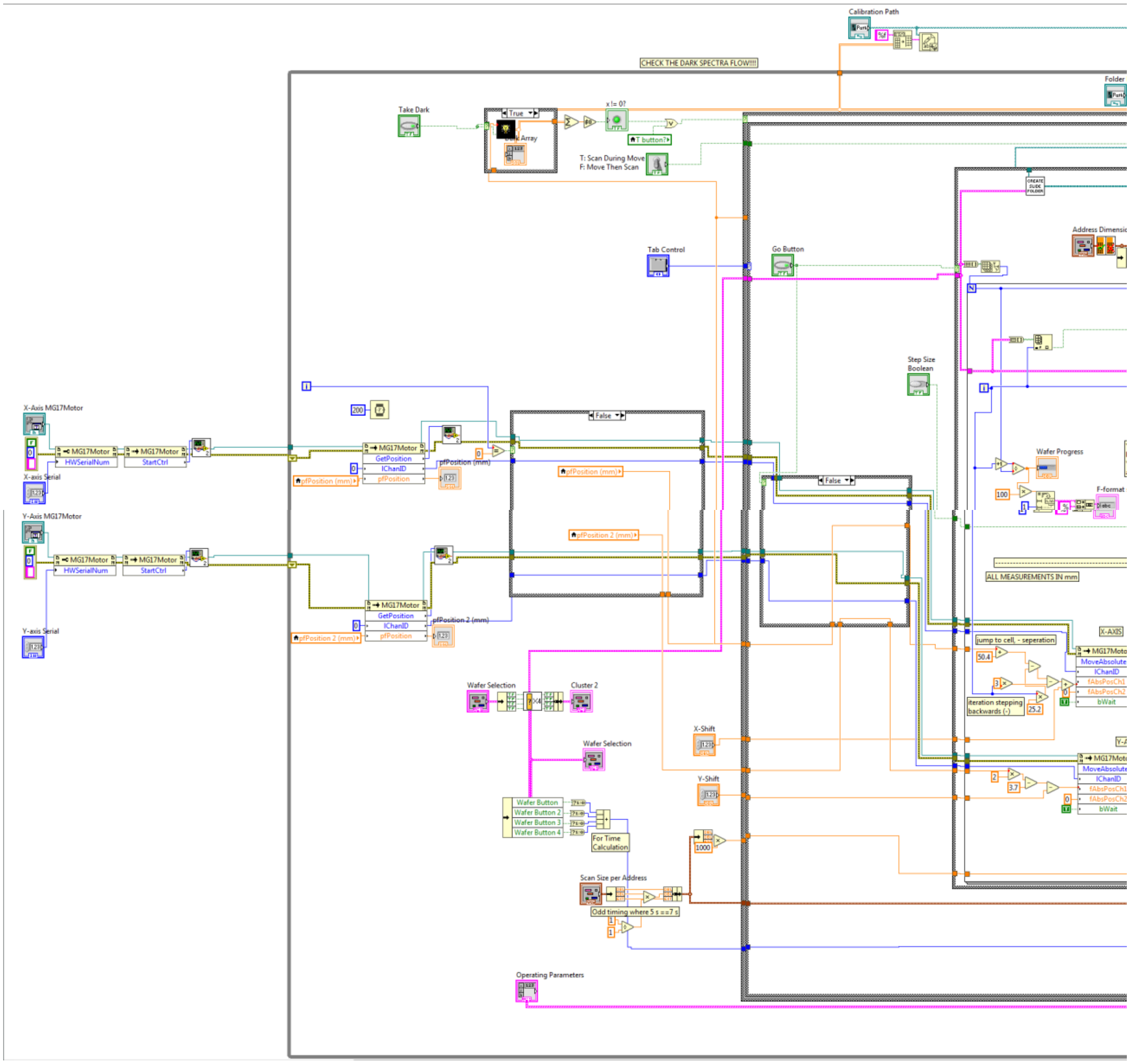

Figure S7: (A) Control code 1/3

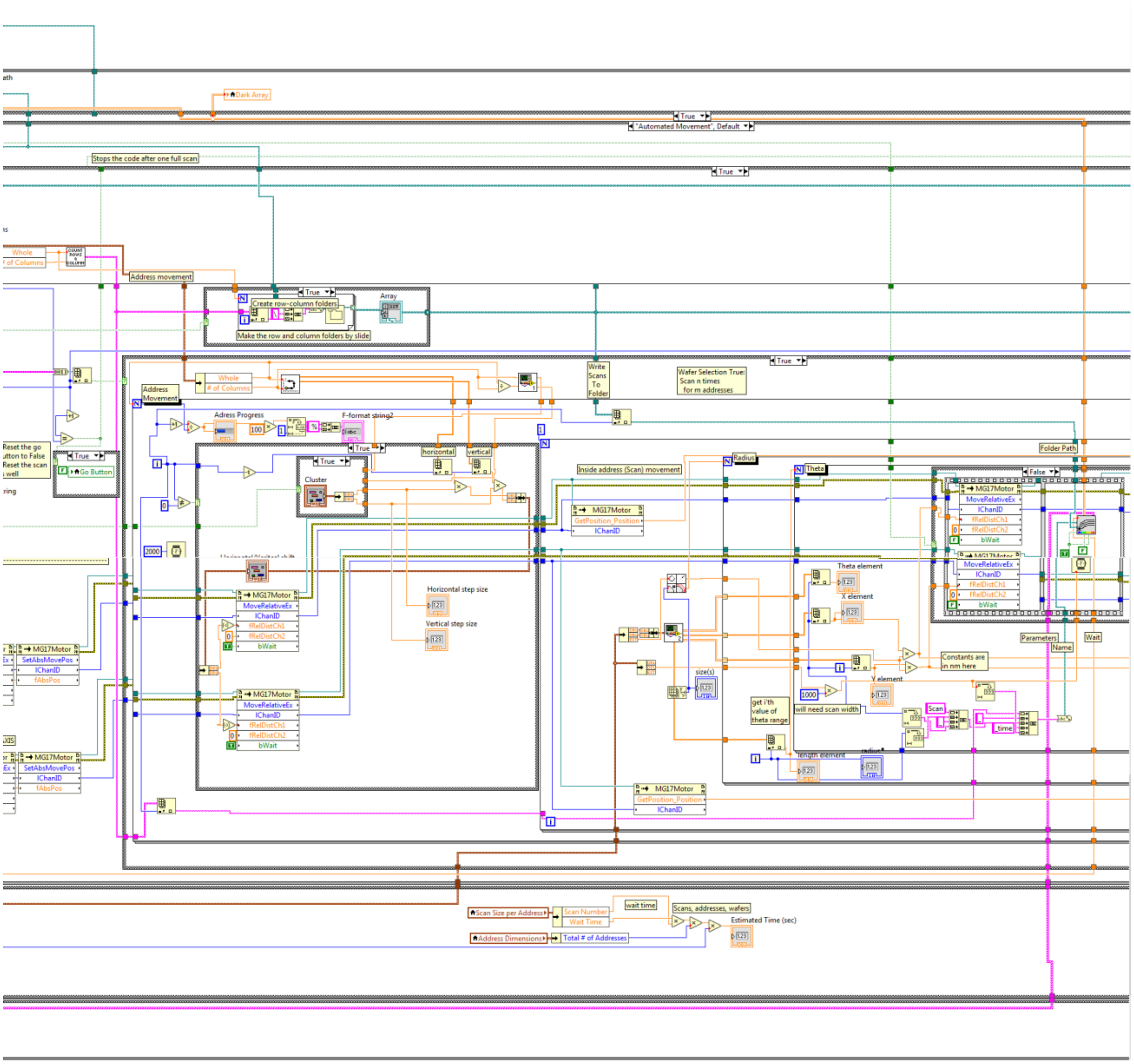

Figure S7: (B) Control code 2/3

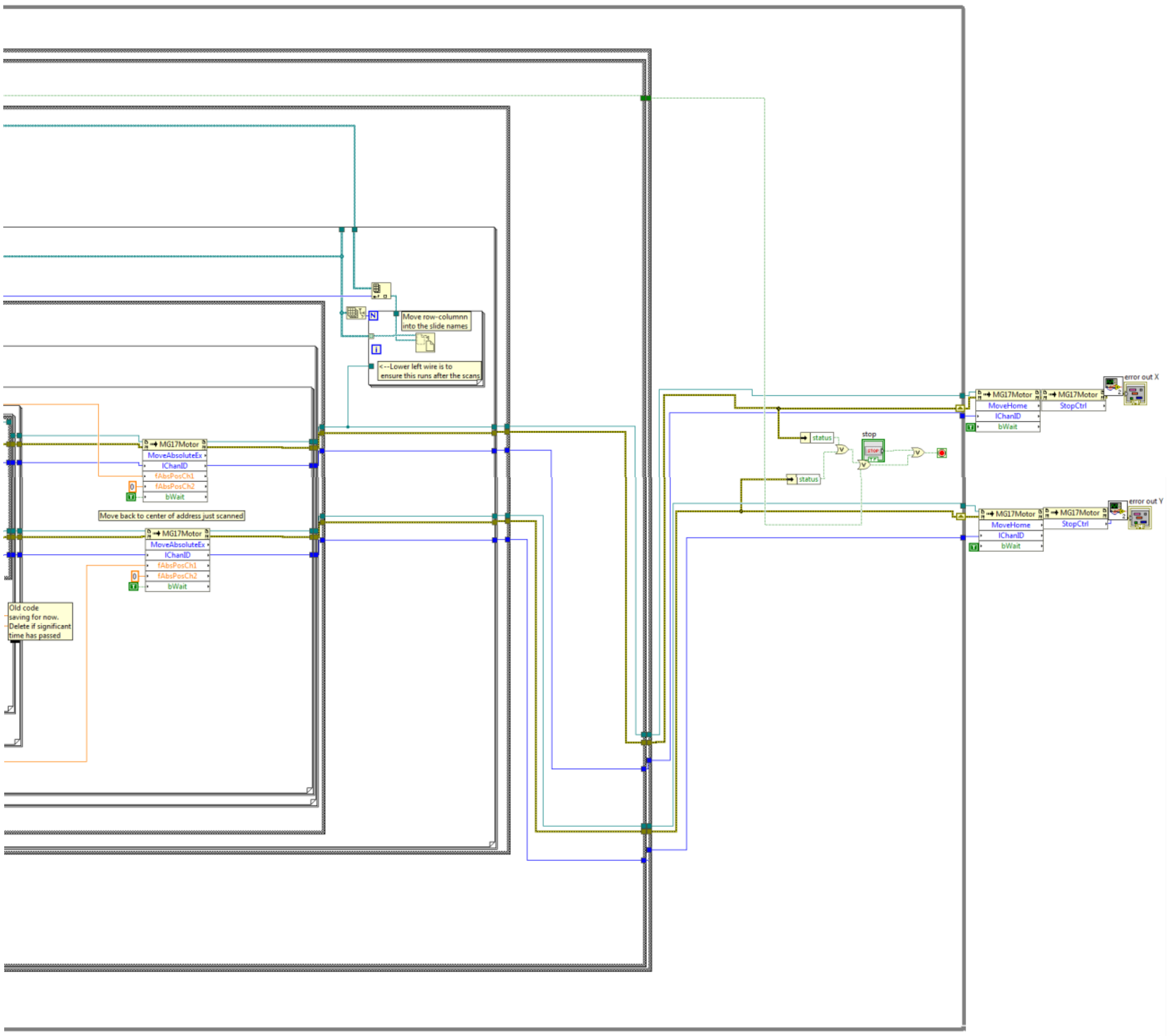

Figure S7: (C) Control code 3/3

After data is collected and saved, a second program is used to analyze the data. First, a file path has to be specified and the file parsed out into a resulting array of values. The code is show in the two part picture below (Figure S8). From top half, left to right: file path and data parsing, peak selecting, maximum height selection, smoothing function, and shoulder finding methods. From bottom half, left to right: Lorentzian model for loop, matching the for of the main peak to the Lorentzian by minimizing the standard deviation of the distances between the Lorentzian and the main peak, flow data out to comparison graphs.

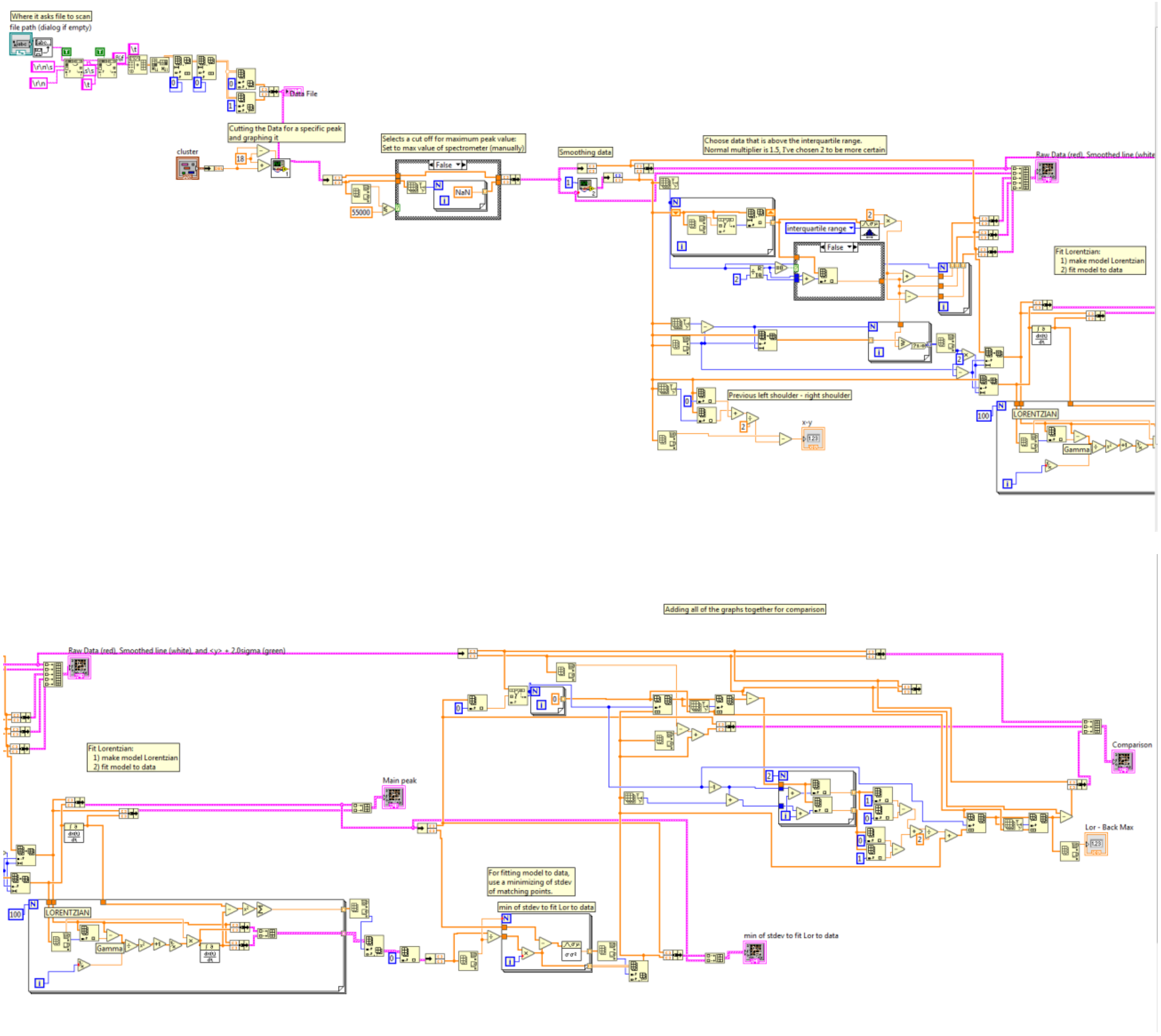

Figure S8: Data analysis program.

The resulting front-end control is depicted in Figure S9. This can either be used as a single file observation of the peak or written as a sub-program for data analysis of a folder with multiple files.

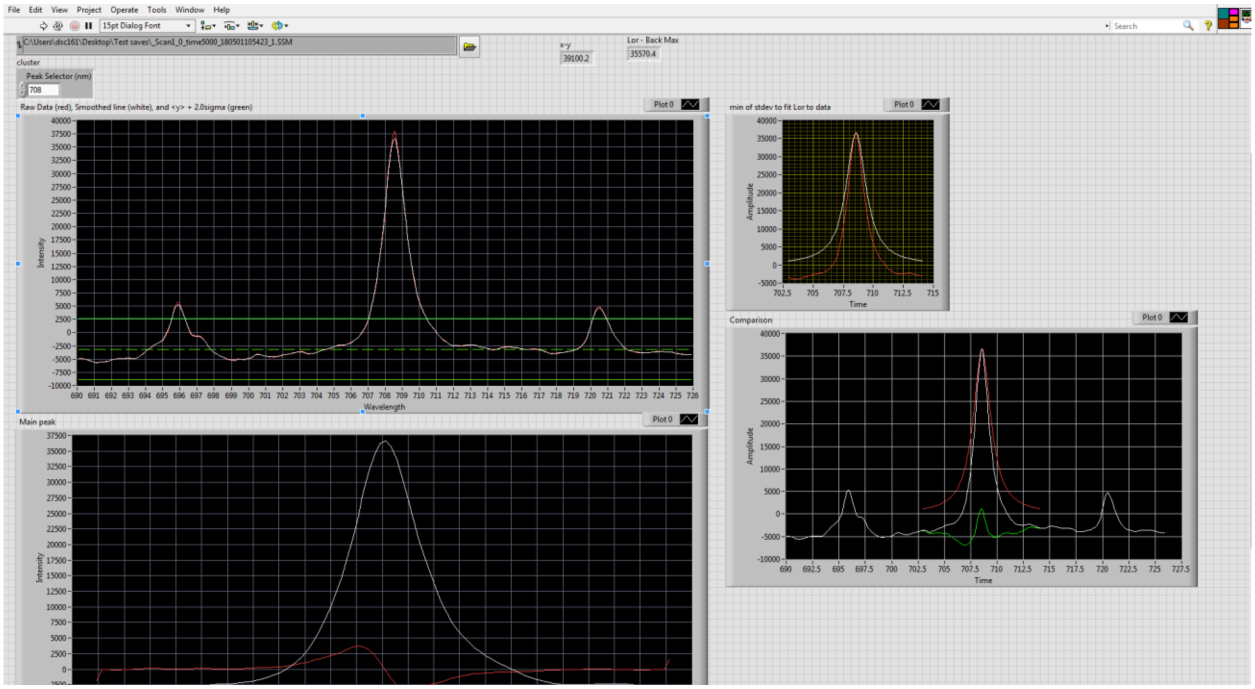

Figure S9: Control panel of the analysis code.

In Figure S9, the left side are cutting and fitting graphs for visualization of what the code is doing. Top left graph depicts the cut off relating to the interquartile range (green line). The program searches around the specified peak and will fit data to the Lorentzian if, and only if, the values are above the interquartile range. Once the interquartile range is crossed the code will end its search. The bottom left graph will the print out the corresponding cut peak (white line) and its derivative (red line).

On the right side of the two large graphs of Figure S9 are a matching Lorentzian to the cut graph and the resulting data. The upper right graph prints out the best fit Lorentzian to the cut data. This is done by a Procrustes method of matching corresponding x and y values of two graphs and minimizing the standard deviation between the two. Once the Lorentzian form is found it is then scaled to the cut data by the ratio of the main peak amplitudes. Once this is done the Lorentzian is cut and printed out on the lower right graph in red. The green line is the resulting subtraction, and the white line is the smoothed data. %One may notice that the green line in the bottom right graph has a bump in it. This is due to the fitting of a continuous line to a discrete data set and subtracting out the corresponding values. This implies that there is a small shift between the data and the fit, but since the bump is consistently below the two side peaks of Raman spectroscopy the approximation is acceptable. After the Lorentzian is found the max value for the peak is the resultant intensity.

3. Acquisition

To acquire the image data, LabView was coupled with SpectraWiz code (provided by StellarNet, Inc. – the manufacturer of Raman Spectrometer). SpectraWiz came with a pre-coded program. The majority of that program was left unchanged, but it was necessary to change some of that code in order to achieve the desired data. The following will be a description and explanation of what was changed in the code.

Below, in Figure S11, is the extended code for the imaging program supplied by SpectraWiz with the modifications for the automated Raman setup. It starts off by initiating the starting files for the SpectraWiz device and passes them into a while loop for imaging purposes. Upon start up the SpectraWiz program first initiates the user interface and then either creates or retrieves spectrometer coefficients and application parameters. Once this is done part of the code is passed to a "Daemon Loop" that will cancel the program at any point in time and also pass the code to imaging parameters, like dwell time. After that the code is then passed into a conditional loop that will either scan, process data (save and write to file), busy (imaging), and exit.

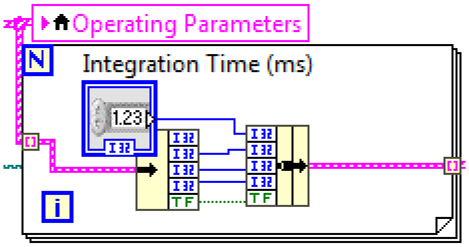

Figure S10: Integration time loop

Modifications were made at getting the dwell time and inside of the conditional loop. The first modification was made to link the wait time of the stage control program to the SpectraWiz program. The wait time control parameter was connected to the SpectraWiz program and then wired up to the starting parameters. In Figure S10, the wait time is called "Integration Time"and is measures in milliseconds. When unbundling the SpectraWiz control parameters, the time the program collects data for is first and therefor at the top of the bundle. Here, the integration time from the stage control was passed into the time auto-filled by SpectraWiz, which allows for the user to control the scanning dwell time.

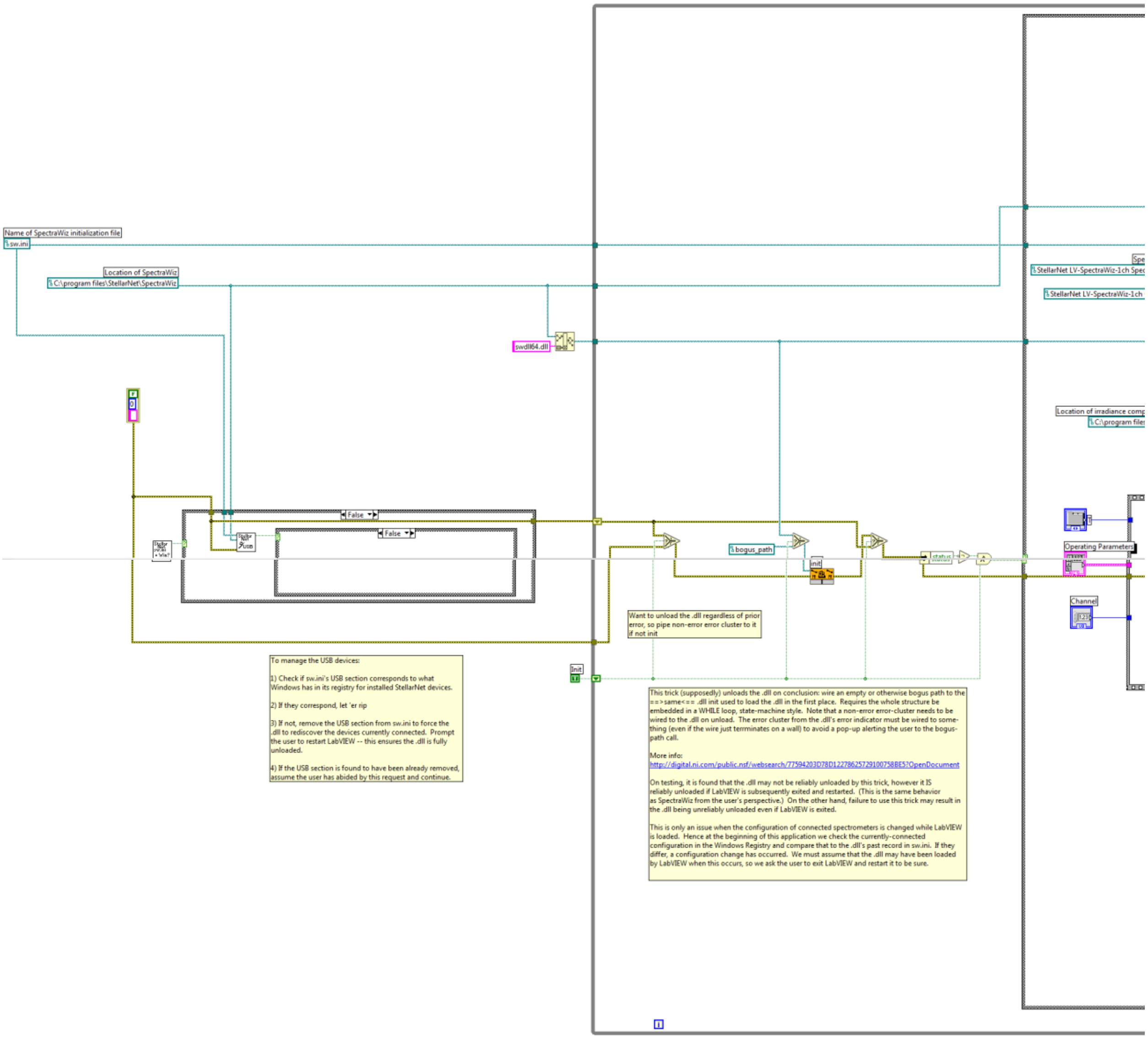

Figure S11: (A) Modified SpectraWiz code 1/3.

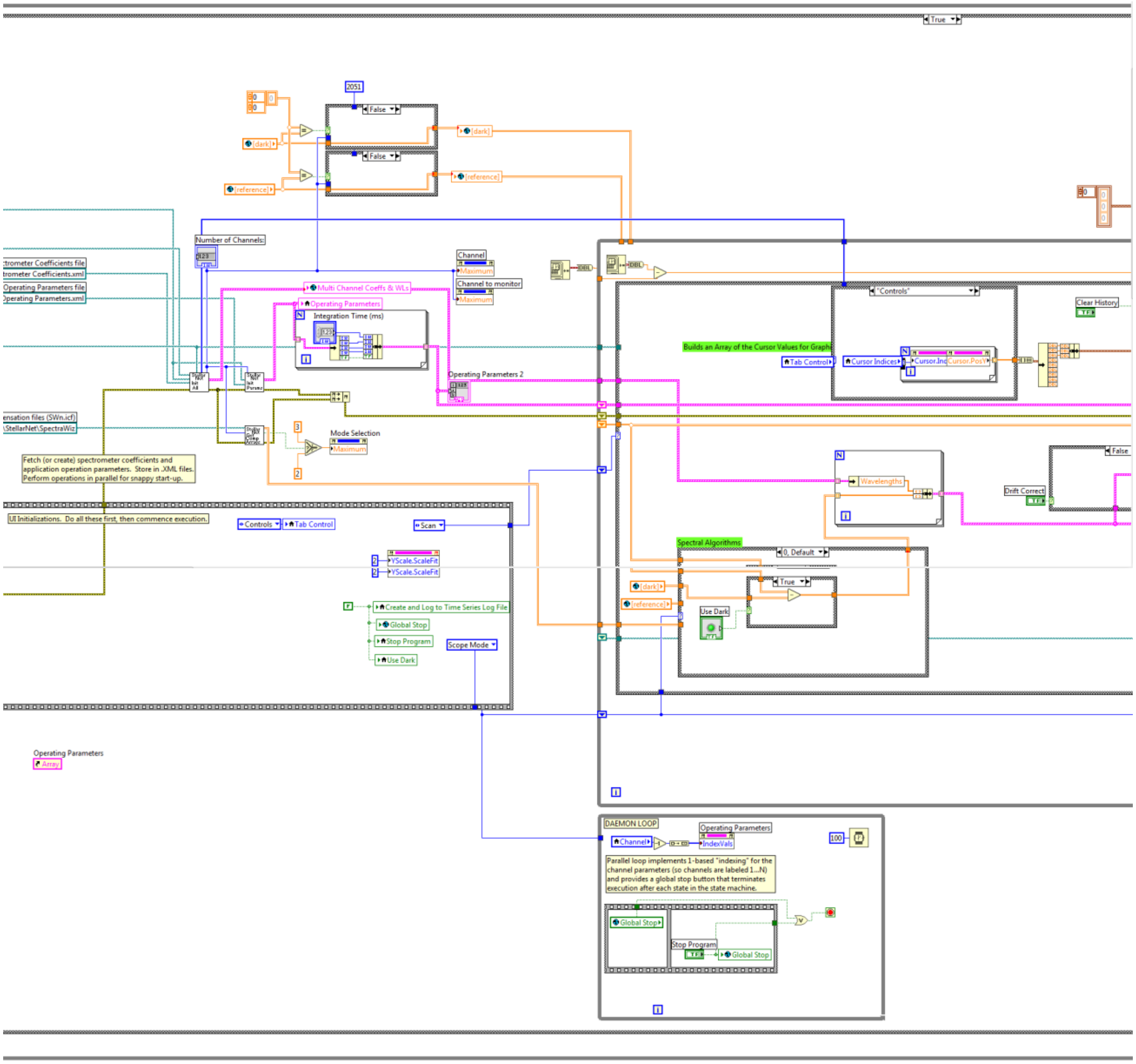

Figure S11: (B) Modified SpectraWiz code 2/3.

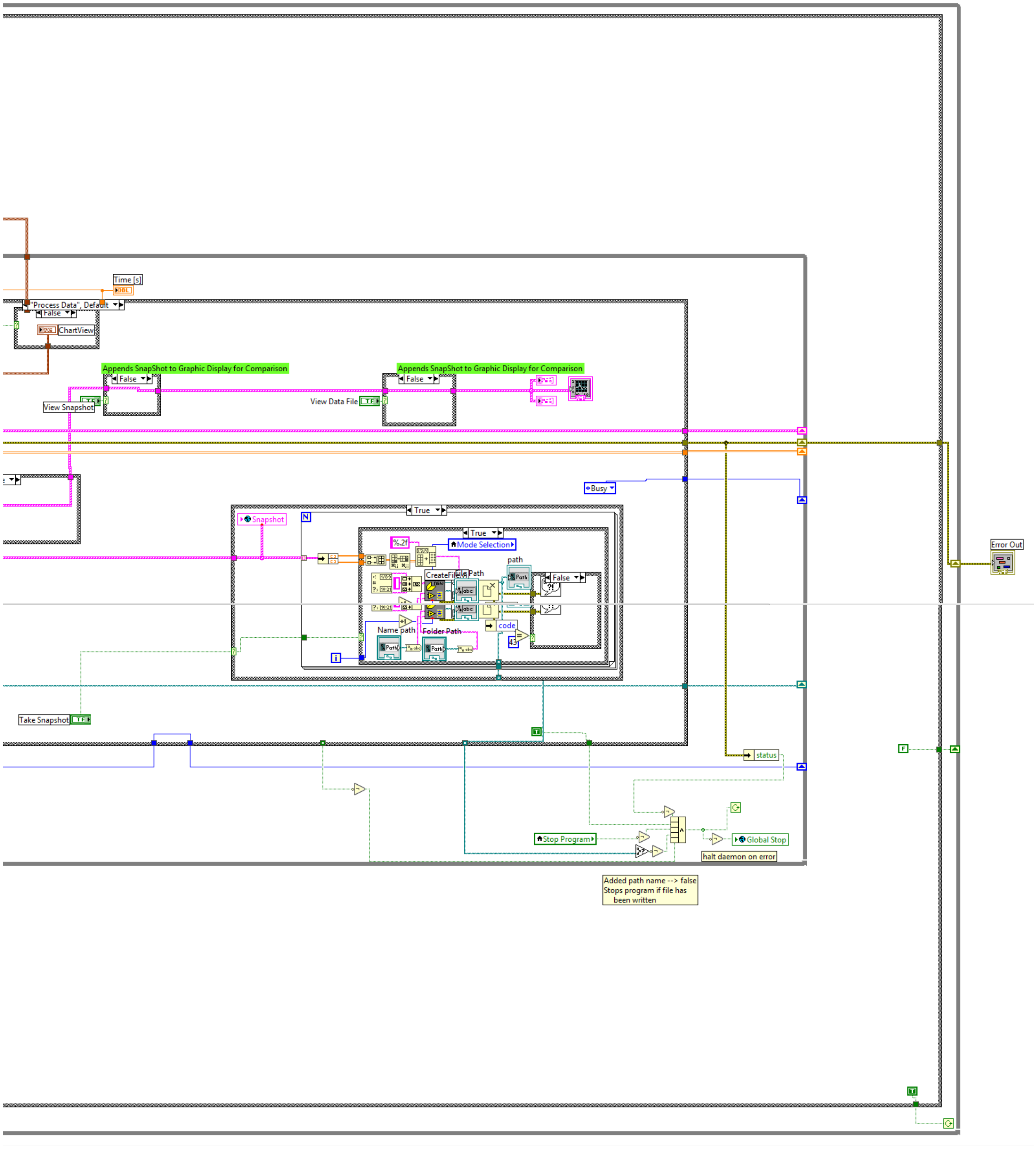

Figure S11: (C) Modified SpectraWiz code 3/3.

After the integration time the code is passed into the conditional loop. Here, the first step is to scan (Figure S12).

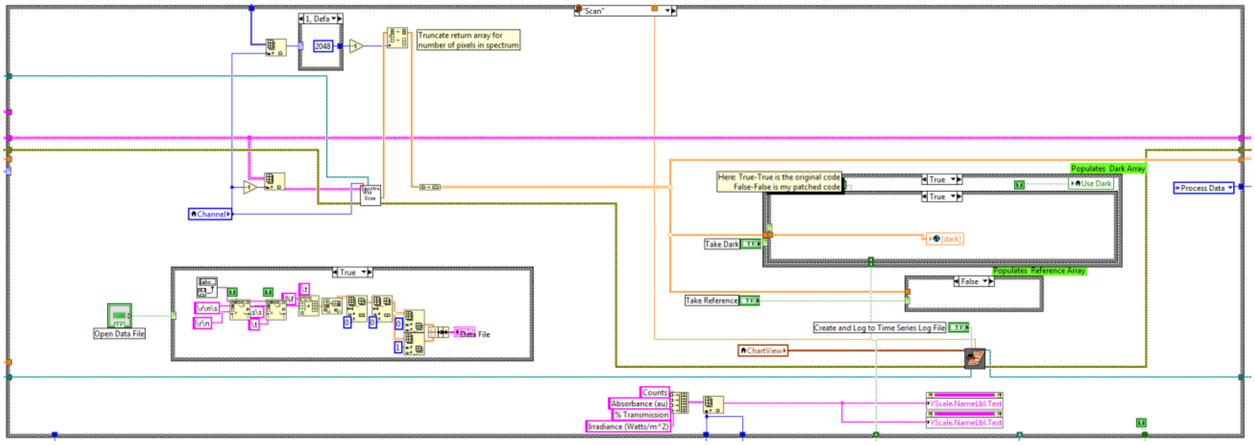

Figure S12: Scan conditional loop

This code was relatively unchanged except for the dark spectra, which was running into complications with communicating between the in-lab made control and the SpecraWiz code. The patch done was to pass the Dark Input from the stage control side into the local variable for the dark spectra of the SpectraWiz program. The use of Boolean true/false loops was employed to keep track of what the original code was when comparing to the patched code. If the two Boolean loops are True-True then it is SpectraWiz's original code. If the Boolean loops are False-True then it is the patched code. Patch work for the dark spectra is shown in Figure S13, which is the right most loop structure in Figure S12.

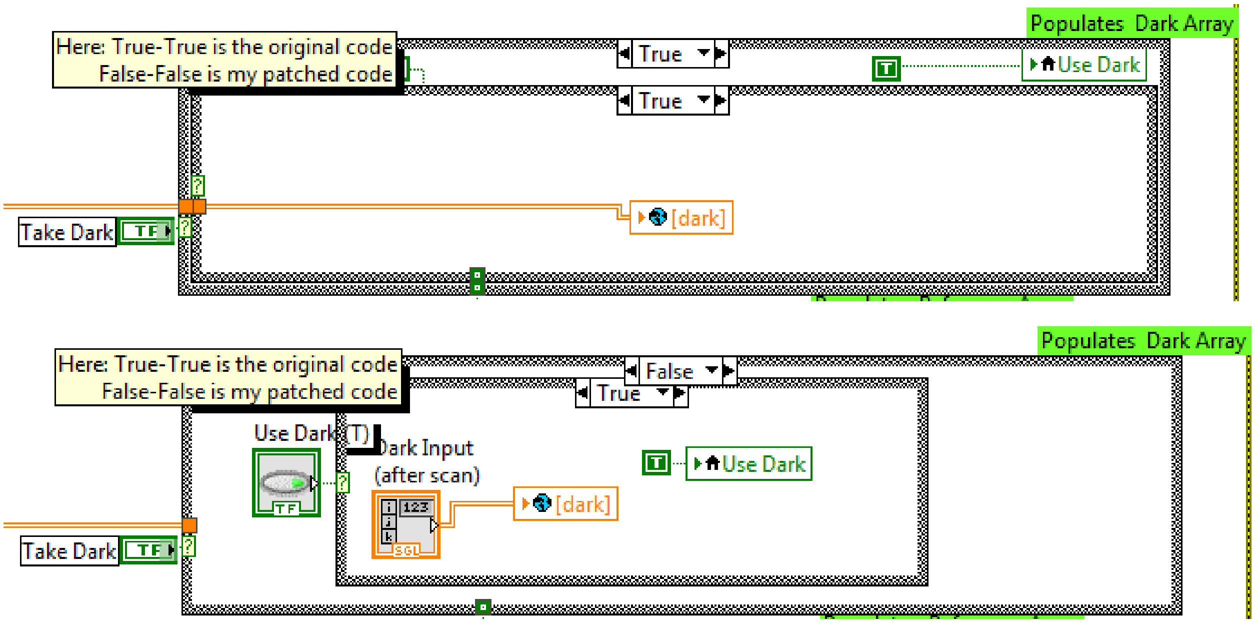

Figure S13: Modified code to work with user controlled dark spectra. Top is the original code and bottom is the code with modifications.

Lastly, when the conditional loop switches to process data, the SpectraWiz code worked perfectly except when trying to name and save files. Because of this a conditional statement was made to check if it was possible to image with and without naming the file. This is the first conditional loop in Figure S14. If the outer loop is false then auto-naming is off and the code should default to the SpectraWiz code. However, since this conditional loop is wired to be true if an image was taken, then auto-naming is on unless one changes the code. Next, is a for loop to drop the array down and a level to work of complexity in order to work with the data. This is then fed into an other conditional loop. Here, the data is given a name based on what address and inner scan number of the program is at, Name path, and saves it to a file with that name, File Path and Create File. The place the data is saved has to be chosen before running the data acquisition so the files can be saved to a folder, this is the Folder Path input. This process is carried out in this inner conditional loop in Figure S14.

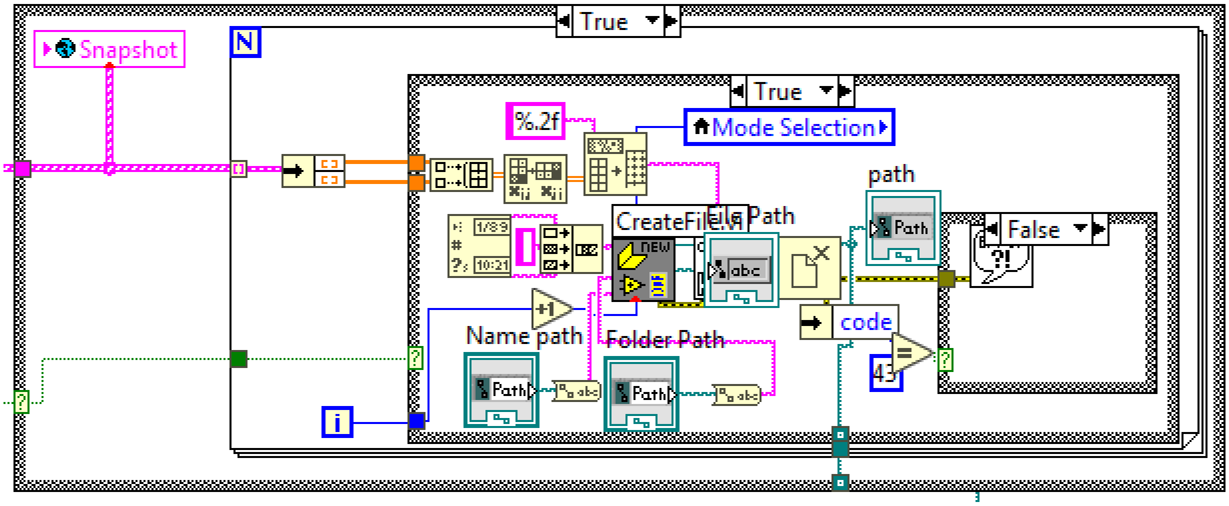

Figure S14: Image to File code.

Additionally, the "Busy" condition will only execute if the program is taking an image and will default to the last command before the program was switch over to the busy condition. The "Exit" condition happens after all other conditions are not met and the stop button is pressed. This will result in closing the program.
